# SIRT1 promotes metastasis of human osteosarcoma cells

**DOI:** 10.18632/oncotarget.12916

**Published:** 2016-10-26

**Authors:** Ning Zhang, Tao Xie, Miao Xian, Yi-Jie Wang, Heng-Yuan Li, Mei-Dan Ying, Zhao-Ming Ye

**Affiliations:** ^1^ Department of Orthopaedics, 2nd Affiliated Hospital, School of Medicine, Zhejiang University, Hangzhou, 310009, P.R. China; ^2^ Institute of Pharmacology and Toxicology, Zhejiang Province Key Laboratory of Anti-Cancer Drug Research, College of Pharmaceutical Sciences, Zhejiang University, Hangzhou, 310058, P.R. China

**Keywords:** osteosarcoma, metastasis, SIRT1, invasion, biomarker

## Abstract

Pulmonary metastasis is the leading cause of mortality in patients with osteosarcoma; however, the underlying mechanism remains unclear. The NAD^+^-dependent deacetylase, sirtuin 1 (SIRT1), has been reported to play a key role in carcinogenesis through deacetylation of important regulatory proteins. Here, we report that SIRT1 promotes osteosarcoma metastasis by regulating the expression of metastatic-associated genes. The SIRT1 protein was significantly upregulated in most primary osteosarcoma tumours, compared with normal tissues, and the SIRT1 expression level may be coupled with metastatic risk in patients with osteosarcoma. Moreover, the results of cell migration and wound-healing assays further suggested that higher expression of SIRT1 promoted invasive activity of osteosarcoma cells. Importantly, downregulating SIRT1 with shRNA inhibited the migration ability of osteosarcoma cells *in vitro* and suppressed tumour lung metastasis in mice. Finally, a gene expression analysis showed that knockdown of SIRT1 profoundly activated translation of its downstream pathway, particularly at migration and invasion. In summary, high levels of SIRT1 may be a biomarker for a high metastatic rate in osteosarcoma patients; inhibiting SIRT1 could be a potent therapeutic intervention for these patients.

## INTRODUCTION

Osteosarcoma is one of the most common primary malignant bone tumours, particularly in children and adolescents [1]. Although conventional therapies have evolved in the past few decades, the prognosis of patients with osteosarcoma remains poor, with a 5-year survival rate of 65% [3]. In addition, 40-50% of patients with osteosarcoma have metastases detectable at diagnosis [3]. Progression of osteosarcoma is thought to be an outcome of cells migrating away from the primary tumour, surviving in circulation, invading lung tissue and establishing metastatic nodules in the lung [4, 5]. Once patients suffer a metastasis, their 5-year survival rate drops to 17% [6]. Therefore, it is of great importance to selectively block the migratory and invasive abilities of osteosarcoma cells. Targeted therapy of key metastatic molecules is an attractive strategy to inhibit tumour metastasis.

Sirtuins are a family of NAD^+^-dependent protein deacetylases that exert multiple cellular functions and are conserved from bacteria to eukaryotes [7]. Silent information regulator 2 (Sir2), the first gene discovered in this family, was originally shown to regulate transcriptional silencing at cell-mating loci, telomeres and ribosomal DNA in yeast [8, 9]. The mammalian sirtuin family consists of seven members, sirtuin 1 (SIRT1) to sirtuin 7 (SIRT7), which share a ~275 amino acid catalytic domain with Sir2 and are suspected to have many similar functions as Sir2 [10]. SIRT1 is the mammalian orthologue most highly related to Sir2 among the seven mammalian sirtuins; it exerts its biological function by deacetylating both histone and non-histone proteins [11, 12]. SIRT1 substrates vary from proto-oncogenes to tumour suppressors, including Myc, p53, nuclear factor kappa beta, Ku70, and forkhead transcription factor [11]. Additionally, overexpression of SIRT1 in tumour cells is correlated with silenced tumour suppressor genes, cancer resistance to chemotherapy and ionising radiation [13].

SIRT1 has been implicated in the cell cycle, as well as apoptosis and cancer metastasis, but its exact role in carcinogenesis remains controversial [14]. Many studies have suggested a role of SIRT1 in tumorigenesis and metastasis [15–18]. In an orthotopic xenograft model of hepatocellular carcinoma (HCC), SIRT1 knockdown resulted in 50% fewer animals developing tumours, and small molecule inhibitor cambinol treatment resulted in an overall lower tumour burden, suggesting that SIRT1 expression positively affects the growth of HCC [10]. SIRT1 overexpression is associated with a higher α-fetoprotein level, higher tumour grade, and absence of a β-catenin mutation [19]. SIRT1 expression predicts poor long-term survival in patients with resected HCC [20]. Silencing SIRT1 also suppresses non-small cell lung cancer (NSCLC) cell proliferation, induces senescence in a p27Kip1-dependent manner and dramatically suppresses tumour formation and proliferation in two distinct NSCLC xenograft mouse models [21]. Some studies show that transgenic Sirt1 expression is oncogenic in murine thyroid and prostate carcinogenesis initiated by Pten-deficiency, and that SIRT1 stabilises the c-MYC protein in cultured thyroid cancer cells [22]. The SIRT1 activator SRT1720 significantly increases the amount of vascular endothelial growth factor secreted by MDA-MB-231 cells and promotes migration of MDA-MB-231 cells. This indicates that SRT1720 promotes the pulmonary metastasis of breast cancer cells, while SIRT1 may be an important target for suppressing metastasis to the lung [23]. Experiments with a mouse model revealed that overexpression of SIRT1 enhanced HCC tumour metastasis *in vivo* and also significantly enhanced the invasive and metastatic potential of HCC cells by inducing the epithelial-mesenchymal transition (EMT) [24]. In addition, SIRT1 knockdown suppresses prostate tumour formation and inhibits metastasis to bone and liver [25]. Another study also showed that reducing SIRT1 expression decreases *in vitro* migration of prostate cancer cells and metastasis in immunodeficient mice, which was largely independent of any general effects of SIRT1 on prostate cancer growth and survival [26].

Interestingly, some studies have claimed that SIRT1 inhibits tumour progression and invasion. Activating SIRT1 inhibits proliferation of Panc-PAUF cells by downregulating cyclin-D1, a β-catenin target molecule [27]. Ectopic overexpression of SIRT1 also greatly reduces proliferation of a human colon cancer cell line, with growth driven by active β-catenin [28]. Knockdown of SIRT1 by short hairpin RNA (shRNA) accelerates tumour xenograft formation in HCT116 cells, whereas SIRT1 overexpression inhibits tumour formation [29]. Reduced SIRT1 levels in HMLER breast cancer cells led to increased metastases in nude mice, and SIRT1 reduces the EMT in cancer and fibrosis by deacetylating Smad4 and repressing the effect of transforming growth factor-β signalling on matrix metalloproteinase-7 (MMP-7), a Smad4 target gene [30]. According to previous studies, it remains controversial whether SIRT1 acts as a tumour promoter or suppressor. In addition, research on Sirt1 in osteosarcoma, particularly osteosarcoma metastasis, remains very limited and there is much that needs to be investigated. To better understand the relationship between SIRT1 and osteosarcoma metastasis, we analysed several primary osteosarcoma tissues from patients and investigated the association between SIRT1 and osteosarcoma metastasis *in vivo* and *in vitro*. Here, we report that SIRT1 modulated osteosarcoma metastasis by regulating expression of metastatic-associated genes. Our study illustrates that high levels of SIRT1 may be a biomarker for a high metastatic rate in patients; furthermore, inhibiting SIRT1 could be a potent therapeutic intervention in patients with osteosarcoma.

## RESULTS

### Osteosarcoma cells are coupled with high expression levels of SIRT1 in vivo

**Figure 1:**
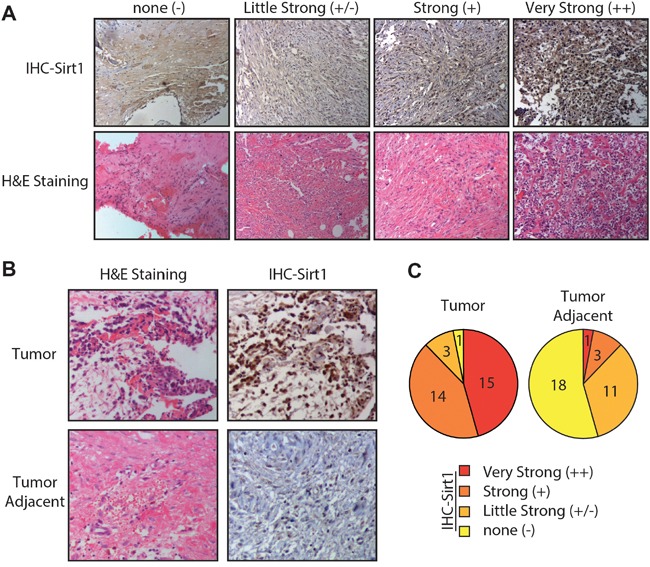
Osteosarcoma cells are associated with high sirtuin 1 (SIRT1) expression levels in vivo **A.** Four representative immunohistochemical analyses of SIRT1 expression levels in human osteosarcoma tissues. Four representative cases were subjected to immunohistochemical staining using an anti-SIRT1 antibody, and cryosections were stained with haematoxylin and eosin. **B.** Representative immunohistochemical analyses of SIRT1 expression levels in human osteosarcoma and adjacent tumour tissues. **C.** SIRT1 expression levels in 33 detected osteosarcoma tumour and adjacent tissue samples were graded and summarised using pie charts. “-”, negative expression; ‘-/+”, slight expression; “+”, strong expression; and “+”, very strong positive expression.

We first evaluated the expression levels of SIRT1 in 33 primary osteosarcoma tissues, and their bone tissues, adjacent to the tumour obtained from patients by immunohistochemistry. The intensity and percentage of staining were determined. The SIRT1 immunohistochemical staining patterns were evaluated by an experienced pathologist and scored as: (1) none (“-”, no positive staining or up to 1% scattered positive cells); (2) slightly strong (“+/− ”, heterogeneous staining, where an area corresponding to at least 20% of the section showed 2–10% positive cells); (3) strong (“+”, heterogeneous, with at least 20% of the section showing 10–50% positive cells); and (4) very strong (“++”, variable to almost homogeneous staining, with at least 20% of the section showing 51–90% positive cells). Figure 1A illustrates four representative osteosarcoma cases with different SIRT1 expression levels. In addition, we found an inverse correlation between SIRT1 expression in different osteosarcoma tissues and their adjacent bone tissues, as indicated in Figure 1B. Therefore, the SIRT1 distributional patterns in all patient samples and the adjacent tumours were analysed: only 1 of 33 osteosarcoma cases (3%) demonstrated no SIRT1 expression in tumour tissues, whereas 29 samples (87.9%) showed intense SIRT1 immunoreactivity. In contrast, in 18 of 33 samples (54.5%) adjacent tumour tissues did not express SIRT1, and only 4 samples (12.1%) showed obvious SIRT1 expression (Figure 1C). Consequently, these results indicate that the SIRT1 expression was significantly upregulated in most osteosarcoma tissues compared with that of normal tissues.

### SIRT1 expression is correlated with osteosarcoma metastasis in vivo

**Figure 2:**
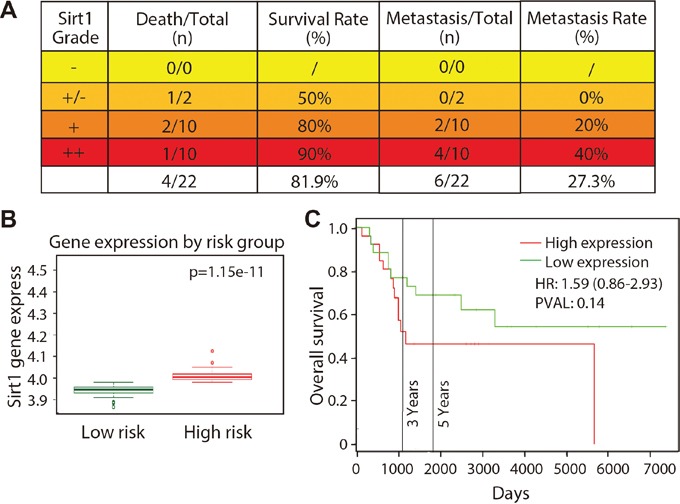
SIRT1 expression is correlated with osteosarcoma metastasis in vivo **A.** Death and metastatic rates of 22 patients with different SIRT1 expression grades were analysed. “-”, negative expression; ‘-/+”, slight expression; “+”, strong expression; and “+”, very strong positive expression. **B.** SIRT1 expression in patients with high- and low-risk osteosarcoma according to the GSE21257 dataset using a SurvExpress analysis. **C.** Relevance between SIRT1 expression level and patient overall survival using PROGgene V2 analysis.

To assess the role of SIRT1 in osteosarcoma cells, we further analysed the survival and metastatic rates of 22 patients with osteosarcoma. As shown in Figure 2A, death rate did not increase in patients who expressed high levels of SIRT1, indicating that SIRT1 expression level may not be associated with survival rate. Interestingly, SIRT1 expression level and metastatic rate were correlated, as the metastatic rate increased from 0% (0/2) in the slightly strong group – and 20% (2/10) in the strong group – to 40% (4/10) in the very strong group. In addition, we also analysed SIRT1 gene expression in the Gene Expression Omnibus datasets. As indicated in Figure 2B, SIRT1 expression was significantly upregulated in high-risk patients with osteosarcoma compared with that of low-risk patients in the GSE21257 dataset using SurvExpress analysis (*p* = 1.15 × 10^−11^). Although a high expression level of SIRT1 appeared to be associated with overall survival, no significant correlation was found (*p* = 0.1412) after the PROGgene V2 analysis (Figure 2C). These results demonstrate that elevated SIRT1 expression level may be associated with high metastatic risk in patients with osteosarcoma.

### Primary osteosarcoma cells expressing higher SIRT1 levels have stronger migration ability

**Figure 3:**
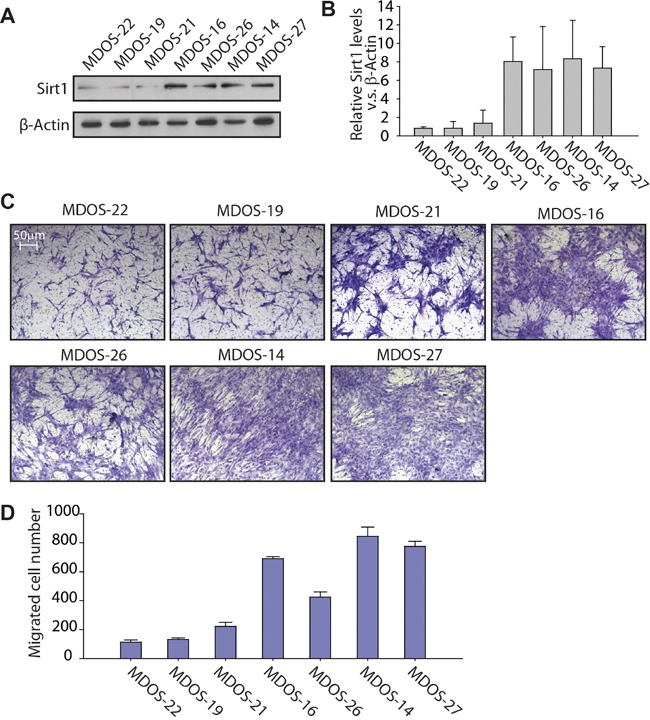
Primary osteosarcoma cells with higher expression of SIRT1 exert stronger migration ability in a Transwell migration assay **A.** Western blotting of SIRT1 and β-actin in the MDOS-22, MDOS-19, MDOS-21, MDOS-16, MDOS-26, MDOS-14 and MDOS-27 primary osteosarcoma cell lines. Anti-SIRT1 and anti-β-actin antibodies were used to detect SIRT1 and β-actin expression, respectively. **B.** Relative SIRT1 protein expression levels in A) were normalised to those of β-actin, as indicated in the histogram. Bars, mean ± standard deviation (SD). **C.** Transwell migration assay of the MDOS-22, MDOS-19, MDOS-21, MDOS-16, MDOS-26, MDOS-14 and MDOS-27 primary osteosarcoma cell lines. Representative images of migrated cells. **D.** The number of migrated cells per field in C) was quantified and is shown as a histogram. Bars, mean ± SD.

To better understand the correlation between SIRT1 expression and the invasion ability of osteosarcoma cells, we chose seven primary osteosarcoma cell samples, cultured from fresh biopsy tissue sections from patients with osteosarcoma, to detect SIRT1 protein expression levels. Our data revealed that three of the seven samples (MDOS-22, MDOS-19 and MDOS-21) expressed a much lower SIRT1 protein level than that in the other four samples (MDOS-16, MDOS-26, MDOS-14 and MDOS-27) (Figure 3A, 3B). The Transwell migration assay was performed to evaluate the invasion ability of these cells. As shown in Figure 3C and 3D, MDOS-16, MDOS-26, MDOS-14 and MDOS-27 cells expressed relative high SIRT1 levels, and very strong invasion ability into the lower chamber of the Transwell, compared with those of low SIRT1-expressing cells (MDOS-22, MDOS-19, and MDOS-21). Therefore, SIRT1 may increase the migration capacity of osteosarcoma cells *in vitro*.

**Figure 4:**
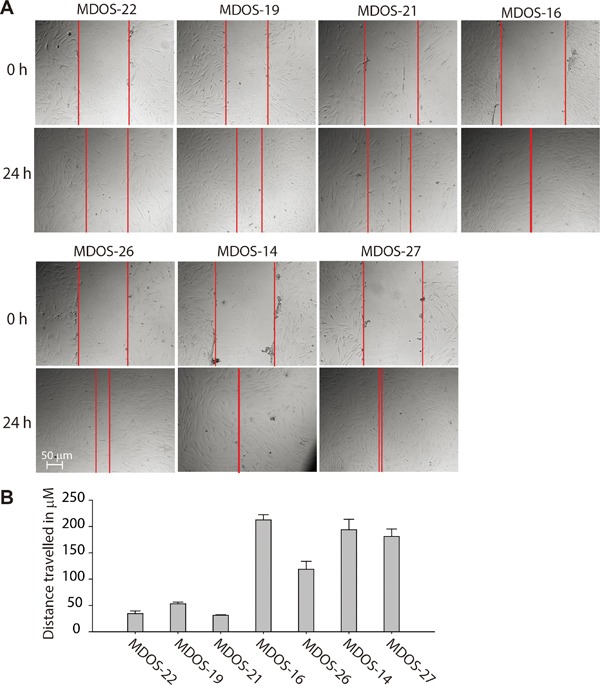
Primary osteosarcoma cells with higher expression of SIRT1 exert stronger invasion ability in the wound healing assay **A.** Nearly confluent MDOS-22, MDOS-19, MDOS-21, MDOS-16, MDOS-26, MDOS-14 and MDOS-27 primary osteosarcoma cells were ‘wounded’ using a 10 μL pipette, and images of the denuded area were taken at 0 and 24 h. **B.** Distance travelled in μM of each cell was measured and shown is as a histogram. Bars, mean ± standard deviation.

Consistent with this finding, the *in vitro* wound-healing assay revealed that primary osteosarcoma cells expressing higher levels of the SIRT1 protein (MDOS-16, MDOS-26, MDOS-14 and MDOS-27) exerted stronger wound-closure capability than those of low SIRT1-expressing cells (MDOS-22, MDOS-19 and MDOS-21) (Figure 4A and 4B). Taken together, these results suggest that high SIRT1 expression is clearly associated with the metastatic potential of human primary osteosarcoma cells.

### SIRT1 knockdown inhibits migration ability of osteosarcoma cells in vitro

**Figure 5:**
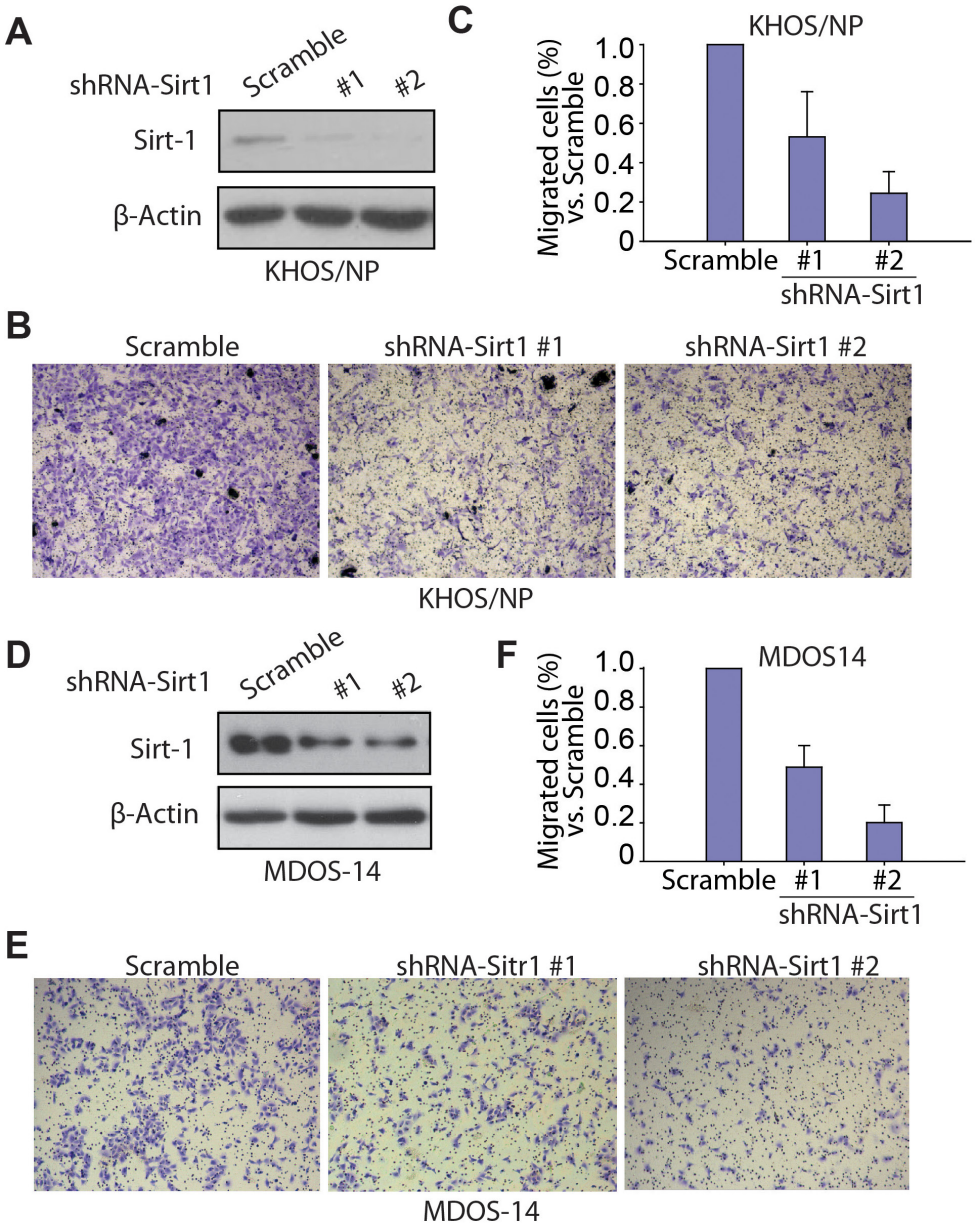
Knockdown of SIRT1 inhibits the migration ability of osteosarcoma cells *in vitro* **A.** Western blotting of SIRT1 expression in KHOS/NP cells after infection with lentivirus- short hairpin RNA (shRNA)-SIRT1 (#1 and #2) or control lentivirus (scramble). **B.** Transwell migration assay of KHOS/NP cells infected with lentivirus-shRNA-SIRT1 (#1 and #2) or control lentivirus (scramble). Representative images of migrated cells are shown. **C.** The number of migrated KHOS/NP cells per field was quantified and is shown as a histogram after normalisation. Bars, mean ± standard deviation (SD). **D.** Western blotting of SIRT1 expression in the MDOS-14 primary osteosarcoma cell line after infection with lentivirus-shRNA-SIRT1 (#1 and #2) or control lentivirus (scramble). **E.** Transwell migration assay of MDOS-14 cells infected with lentivirus-shRNA-SIRT1 (#1 and #2) or control lentivirus (scramble). Representative images of migrated cells are shown. (C) **F.** The number of migrated MDOS-14 cells per field was quantified and is shown as a histogram after normalisation. Bars, mean ± SD.

To further address the effect and importance of SIRT1 in osteosarcoma cell migration and metastasis, we knocked down SIRT1 protein expression in osteosarcoma cells using shRNA. The KHOS/NP osteosarcoma cell line and the MDOS-14 primary osteosarcoma blast line were used in our study. Lentiviral transduction enabled stable downregulation of SIRT1 compared with that in vector-transduced cells in the KHOS/NP osteosarcoma cell line (Figure 5A). The Transwell migration assay showed that shRNA-SIRT1 (#1 and #2)-transduced KHOS/NP cells migrated less efficiently into the lower chamber of the Transwell compared with scrambled shRNA transduced control cells (Figure 5B and 5C). Similar results occurred in the MDOS-14 primary osteosarcoma cell line. Two specific sequences against SIRT1 significantly inhibited endogenous SIRT1 expression in MDOS-14 cells (Figure 5D), and SIRT1 knockdown clearly inhibited the invasion ability of primary cells (Figure 5E and 5F). Moreover, we also performed knockdown experiments in another two cell lines (HOS and U2OS) and overexpression experiments in three different osteosarcoma cell lines (HOS, U2OS and KHOS/NP cells). Similar results were observed. Overexpression of SIRT1 increases the migration ability of KHOS/NP, HOS and U2OS cells (Supplementary Figure S2). Inversely, down-regulation of SIRT1 inhibits the migration of HOS and U2OS cells (Supplementary Figure S3A-S3C). Therefore, our data suggest that the SIRT1 protein is required for migration of osteosarcoma cells *in vitro*.

### Depleting SIRT1 reduces lung metastasis of osteosarcoma cells in mice

**Figure 6:**
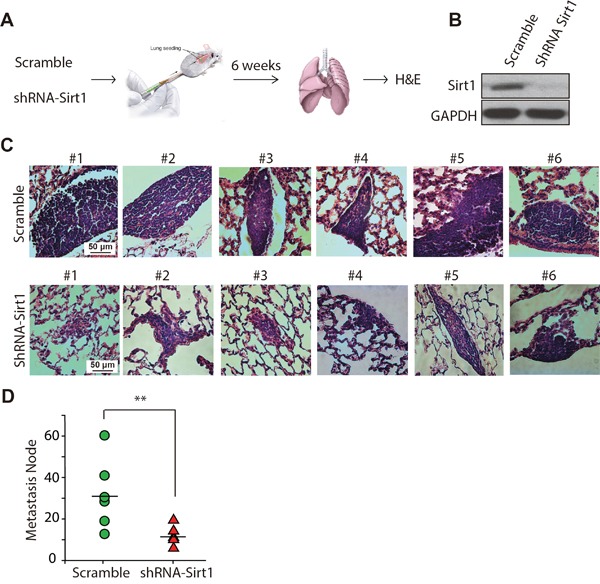
Knockdown of SIRT1 reduces lung metastasis of osteosarcoma cells *in vivo* **A.** KHOS/NP cells infected with lentivirus-shRNA-SIRT1 (#2) or control lentivirus (scrambled) were injected into the tail vein of BALB/c (nu/nu) mice (n = 6), and formation of metastatic nodes was determined at week 6. **B.** Western blotting of SIRT1 expression in KHOS/NP cells after infection with lentivirus-shRNA-SIRT1 (#2) or control lentivirus (scramble). **C.** Representative haematoxylin and eosin staining of lung tissue is shown. **D.** The number of metastatic nodes per lung is shown. Bars represent mean ± standard deviation (n = 6). **, p < 0.01.

To further verify the effects of SIRT1 on migration and metastasis of KHOS/NP cells *in vivo*, we performed tail-vein xenografts in BALB/c (nu/nu) mice and examined the rates of lung colonisation. The flow chart for the experiment is displayed in Figure 6A. Scrambled or shRNA-SIRT1-transduced KHOS/NP cells (1 × 10^5^) were injected intravenously into nude mice (Figure 6B). The mice were sacrificed 6 weeks later, and lung metastatic nodes were detected by H&E staining. In agreement with the *in vitro* results, the histological examination of lung tissues revealed that downregulating SIRT1 strongly reduced the number and size of lung metastatic nodes. Representative cases of H&E staining of lung from each mouse are shown in Figure 6C. As the data show, SIRT1 knockdown decreased the size of the lung metastatic nodes compared to that in the control group. Mice injected with SIRT1 knockdown cells formed a mean of only 10 metastatic nodes, whereas mice injected with control cells formed 10-60 metastatic nodes per lung (*p* < 0.01; Figure 6D). Consistent with our *in vitro* data, SIRT1 knockdown inhibited lung metastasis of osteosarcoma cells *in vivo*.

### Genomic changes in KHOS/NP cells following SIRT1 downregulation

**Figure 7:**
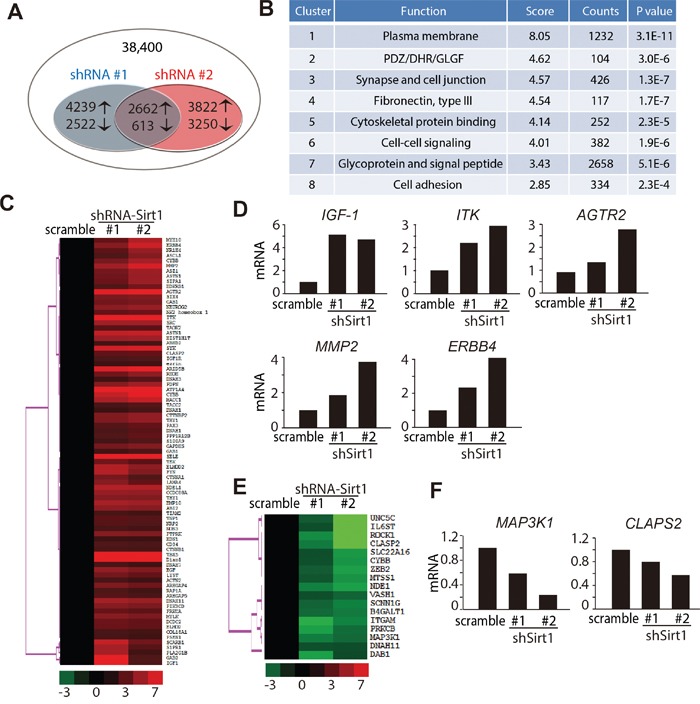
Genome changes in KHOS/NP cells following downregulation of SIRT1 **A.** Schematic representation comparing the gene expression profiles in KHOS/NP cells. Overlapping smaller circles reflect the 3,275 shared genes (2,662 upregulated and 613 downregulated) induced by lentivirus-shRNA-SIRT1 (#1 and #2). **B.** Functional annotation clustering of the 3,275 overlapped genes according to their DAVID enrichment score. A higher enrichment score for a group indicates that the gene members in the group are involved in more important terms. **C, E.** Heatmap display of hierarchical clustering of overlapped genes sorted from Table 1. A total of 100 genes associated with metastasis whose expression changed ≥ two-fold were clustered. **D, F.** Real-time polymerase chain reaction analysis was used to validate five upregulated D) and two downregulated F) genes in KHOS/NP cells.

Our investigation revealed that SIRT1 was crucial for osteosarcoma cells to migrate, and that deleting SIRT1 efficiently inhibited invasion of osteosarcoma cells. SIRT1 deacetylates histones, as well as a broad range of transcription factors and co-regulators, thereby regulating target gene expression [31, 32]. To gain insight into the mechanism of SIRT1 knockdown-induced inhibition of osteosarcoma cell migration, we performed a gene expression analysis with RNA extracted from KHOS/NP cells after they were transduced with shRNAs targeting SIRT1 (#1 and #2) or the scrambled control shRNA. Genes up- or downregulated by more than two-fold in the shRNA-SIRT1 groups were selected. Among these 38,400 transcripts, 3,275 (2,662 upregulated and 613 downregulated) genes displayed significant changes (Figure 7A). A David functional annotation clustering analysis further revealed that these altered genes were mainly involved in plasma membrane (1232/3,275), PDZ/DHR/GLGF (104/3,275), synapse and cell junction (426/3,275), fibronectin III (117/3,275), cytoskeletal protein binding (252/3275), cell-cell signalling (382/3275), glycoprotein and signal peptide (2658/3,275) and cell adhesion (334/3,275) (Figure 7B).

The expression of metastatic-associated genes was further analysed, and 100 genes involved in cell metastasis were identified (Table 1). These 100 altered genes were visualised with TreeView; 83 were identified as upregulated and 17 as downregulated (Figure 7C and 7E). Real-time polymerase chain reaction analysis was conducted to confirm the array results of several altered genes that regulated metastasis. As illustrated in Figure 7D and Supplementary Figure S3D, the expression of *insulin-like growth factor-1*, *ITK*, *angiotensin II type 2 receptor*, *MMP2* and *ERBB4* were significantly upregulated after depleting SIRT1. In contrast, the expression of *mitogen-activated protein kinase kinase kinase 1* and *CLAPS2* were obviously downregulated in SIRT1 knockdown cells (Figure 7F and Supplementary Figure S3D). Taken together, these data strongly indicate that SIRT1 knockdown has a profound activating effect on the translation of its downstream pathway, particularly that for migration and invasion.

**Table 1:** Sirt1 array genes

Gene ID	Gene Symbol	log 2 (scramble vs. scramble)	log 2 (shRNA Sirt1 #1 vs. scramble)	log 2 (shRNA Sirt1 #2 vs. scramble)
206211_at	SELE	0	7.092691	5.894409
1564241_at	ATP1A4	0	4.090978	4.776326
233377_at	ARID5B	0	3.545219	4.431391
211339_s_at	ITK	0	4.032259	4.103357
1557865_at	Diap1	0	4.548669	4.067227
1563018_at	TBX5	0	4.464877	4.005566
222321_at	AGTR2	0	3.636885	3.88906
203923_s_at	CYBB	0	3.176981	3.715001
244023_at	SYK	0	3.379857	3.504922
1566677_at	MMP2	0	2.580377	3.496527
214053_at	ERBB4	0	2.120652	3.319697
213197_at	ASTN1	0	3.099482	3.19205
1559361_at	MACC1	0	2.720081	3.170157
227553_at	NDEL1	0	2.920382	2.862516
237491_at	MYH10	0	1.609199	2.825717
217431_x_at	CYBB	0	1.915849	2.651022
1558210_at	SRC	0	2.606417	2.628936
204879_at	PDPN	0	2.1307	2.592036
1553568_a_at	HIST1H1T	0	2.501108	2.567836
208292_at	BMP10	0	2.713226	2.560215
1569729_a_at	ASZ1	0	1.857179	2.520975
216796_s_at	SIPA1	0	1.716702	2.482839
237804_at	DNAH11	0	2.650658	2.464855
1560900_a_at	ASTN1	0	1.790438	2.448615
215632_at	NEUROG2	0	2.229938	2.443174
243799_x_at	NR1H4	0	1.520381	2.406532
219387_at	CCDC88A	0	2.387466	2.358463
242769_at	CTTNBP2	0	2.047317	2.342755
208851_s_at	THY1	0	2.031299	2.334322
1557837_a_at	ELMOD2	0	2.887447	2.29459
216168_at	RHOH	0	1.782789	2.267731
216113_at	ABI2	0	2.277083	2.13853
209987_s_at	ASCL1	0	1.275635	2.104959
207116_s_at	GAPDHS	0	1.871879	2.095805
243006_at	FYN	0	2.923039	2.073735
213869_x_at	THY1	0	2.093254	2.068773
1568898_at	PTPRK	0	2.130188	2.064466
231797_at	SIX4	0	1.946596	2.059131
206254_at	EGF	0	2.355848	2.046819
1569956_at	MYLK	0	2.179593	2.005815
211230_s_at	PIK3CD	0	2.136025	1.979493
226002_at	GAB1	0	1.809276	1.918858
207510_at	BDKRB1	0	1.270388	1.822256
215835_at	SCARB1	0	3.159161	1.8069
241003_at	ARHGAP4	0	2.037725	1.78245
231315_at	NK2 homeobox 1	0	1.574108	1.713181
1562511_at	LYST	0	1.839708	1.597401
216059_at	PAX3	0	1.383118	1.590649
238224_at	CLASP2	0	1.53692	1.589905
222926_at	DCDC2	0	1.727901	1.589103
233700_at	PPP1R12B	0	1.353101	1.567755
231113_at	LAMA4	0	2.133655	1.563041
1554857_at	ELMO2	0	1.693918	1.561817
1555939_at	PRKCA	0	1.647023	1.49706
217711_at	TEK	0	1.781049	1.477221
204878_s_at	TAOK2	0	1.458521	1.473092
1567378_x_at	DNAH1	0	1.251449	1.439851
204642_at	S1PR1	0	2.669766	1.429096
1570025_at	TACC2	0	1.194575	1.401975
206568_at	TNP1	0	1.471619	1.395182
1560803_at	DNAH3	0	1.094843	1.390687
232701_at	NRP2	0	1.410842	1.337749
203535_at	S100A9	0	1.149642	1.331771
1566958_at	GAB2	0	3.969119	1.326655
236934_at	IGF1R	0	1.249829	1.306312
209541_at	IGF1	0	4.868146	1.293599
217366_at	CTNNA1	0	1.871555	1.285494
209543_s_at	CD34	0	1.320598	1.26174
214987_at	GAB1	0	1.105924	1.25405
242110_at	ARHGAP5	0	1.409215	1.231669
228112_at	DNAH1	0	1.00748	1.198105
242558_at	CTNNB1	0	1.232711	1.184166
87100_at	ABHD2	0	1.13774	1.165258
205581_s_at	NOS3	0	1.214836	1.149663
1568732_at	COL18A1	0	1.230828	1.134776
239409_at	RAP1A	0	1.281091	1.120755
206311_s_at	PLA2G1B	0	2.66801	1.111966
219950_s_at	TIAM2	0	1.182572	1.110632
238645_at	ezrin	0	1.045305	1.089676
234476_at	DNAH7	0	1.206944	1.080146
242875_at	PSEN1	0	1.164353	1.066398
203863_at	ACTN2	0	1.206237	1.044383
1564630_at	EDN1	0	1.05669	1.026215
1555341_at	UNC5C	0	−1.24853	−4.67161
1569981_at	ROCK1	0	−1.94717	−4.14226
238048_at	CLASP2	0	−1.70679	−3.57893
234474_x_at	IL6ST	0	−1.07681	−3.47674
227843_at	NDE1	0	−1.78393	−2.23082
233031_at	LOC100128821 /// ZEB2	0	−1.26682	−2.21123
1561864_at	SLC22A16	0	−1.09064	−2.1212
233538_s_at	CYBB	0	−1.10286	−2.06586
1556192_x_at	MTSS1	0	−1.17339	−2.01977
205786_s_at	ITGAM	0	−2.47543	−1.82788
241436_at	SCNN1G	0	−1.36877	−1.79371
216627_s_at	B4GALT1	0	−1.4641	−1.6739
227824_at	PRKCB	0	−2.23079	−1.49389
214786_at	MAP3K1	0	−1.7433	−1.43081
242566_at	VASH1	0	−1.09525	−1.37028
1553159_at	DNAH11	0	−1.10406	−1.06759
242840_at	DAB1	0	−2.25233	−1.01374

## DISCUSSION

Pulmonary metastasis has been recognised as the main cause of fatal outcomes in patients with osteosarcoma, but its molecular mechanism is rarely discussed [33]. In this study, we evaluated SIRT1 expression level in different clinical samples. Most osteosarcoma tumour tissues showed strong or very strong SIRT1 expression, whereas more than half of the adjacent tumour tissue samples did not express SIRT1. The correlation between SIRT1 expression and malignant tumours has been reported previously. Immunohistochemical expression of SIRT1 was evaluated in patients with diffuse large B-cell lymphoma (DLBCL), using a 2 mm tissue microarray core, and SIRT1 was expressed in 74% (77/104) of patients [34]. SIRT1 expression levels, in normal and breast tumour tissues from 28 patients with breast cancer, were evaluated to determine correlations with clinicopathological variables. These results also showed that SIRT1 expression was higher in tumour tissues than in matched normal tissues at the protein level, but not at the transcriptional level [35]. Taken together, SIRT1 may participate in tumour formation and progression in many different kinds of cancers. Furthermore, we analysed the association between SIRT1 expression and progression in patients with osteosarcoma; stronger SIRT1 expression was significantly correlated with a higher metastatic rate. A previous study reported that SIRT1 mRNA and protein are overexpressed in pancreatic cancer tissues, and increased SIRT1 expression was correlated with tumours from patients > 60 years of age, or with tumours > 4 cm, a higher TNM stage or the presence of lymph node or hepatic metastases [36]. We reported for the first time the association between increased SIRT1 expression and poor prognosis in patients with osteosarcoma. Moreover, SIRT1 may be a biomarker for diagnosing and predicting osteosarcoma metastasis.

Further experiments were conducted to determine whether SIRT1 regulates the migration ability of osteosarcoma. Three of the seven primary cell lines tested demonstrated much lower SIRT1 protein expression than that of the other four (Figures 3A and B). As expected, the three cell lines with lower SIRT1 expression demonstrated an obviously lower capacity for invasion and migration in the Transwell migration and wound healing assays (Figures 3D and 4). These results revealed the notable correlation between SIRT1 protein level and osteosarcoma metastatic ability, consistent with the analysis of patient progression. Interestingly, we also found that SIRT1 expression was not related to the sensitivity of osteosarcoma cells to chemotherapy (Supplementary Figure S1). To confirm the key function of SIRT1 in osteosarcoma metastasis, we knocked down SIRT1 in the KHOS/NP osteosarcoma cell line and MDOS-14 primary cells. In line with our expectations, SIRT1 knockdown significantly reduced the ability of both cell types to migrate. Furthermore, a nude mice lung-metastasis model verified that deleting SIRT1 enabled a lower metastasis rate *in vivo* (Figures 5 and 6). Thus, we report a crucial function of SIRT1 in osteosarcoma cell metastasis.

Although our results revealed that SIRT1 promoted invasion of osteosarcoma, several other studies have claimed that SIRT1 inhibits tumour progression. SIRT1 deacetylates β-catenin, suppresses its ability to activate transcription, drives cell proliferation and inhibits intestinal tumour formation in patients with colon cancer [28]. Moreover, inconsistent with our finding that stronger SIRT1 expression was coupled with a higher metastatic rate, another study reported that SIRT1 was overexpressed in 25% of stage I/II/III colorectal adenocarcinomas, but was rarely found in advanced stage IV tumours; meanwhile, 30% of carcinomas showed lower than normal SIRT1 expression [29]. These inconsistent results appear to be due to the different characteristics of different tumours. No report has stated that SIRT1 inhibits tumour progression or metastasis. Therefore, our study is the first to reveal the important role of SIRT1 in the metastatic potential of osteosarcoma cells.

In summary, our study revealed, for the first time, a positive correlation between SIRT1 expression and metastatic rate in patients with osteosarcoma. Our data provide evidence for an important role of SIRT1 in promoting the metastasis of osteosarcoma, by regulating transcription of targeted genes. Therefore, SIRT1 deserves further study as a potential biomarker for diagnosing and predicting osteosarcoma metastasis. Our study also indicates a new opportunity to treat osteosarcoma metastasis by targeting SIRT1.

## MATERIALS AND METHODS

### Cell lines and plasmids

The KHOS/NP cell line was kindly provided by Dr. Lingtao Wu (University of Southern California, Los Angeles, CA, USA). All primary osteosarcoma blasts were from fresh biopsy tissue sections from patients with osteosarcoma, as described previously [37, 38]. All cells were cultured in DMEM or RPMI1640 medium supplemented with 10% foetal bovine serum (FBS) in a humidified atmosphere of 5% CO_2_ at 37°C.

### Human tissue specimens

Clinical samples were obtained from patients with osteosarcoma at the Second Affiliated Hospital of Zhejiang University, School of Medicine. Written informed consent from patients, and approval from the Institutional Research Ethics Committee of the hospital, were obtained prior to the use of their clinical materials for research purposes.

### Lentivirus transduction

The shRNA-expressing lentiviral vector pGFP-V-RS against the SIRT1 gene was obtained from Origene (cat. #: TG309433; Rockville, MD, USA). The virus particles were harvested 48 h after transfecting 293FT cells. The cells were grown in 6-well plates at 60–70% confluency, and 1 mL of viral supernatant was added with 1 μL Polybrene for a stable transfection.

### Western blotting

Western blotting was conducted as reported previously. Antibody against SIRT1 was purchased from Cell Signaling Technology (Danvers, MA, USA). Antibody against β-actin was purchased from Santa Cruz Biotechnology (Santa Cruz, CA, USA). Western blots were visualised with horseradish peroxidase (HRP)-conjugated secondary antibodies (Jackson ImmunoResearch Laboratories Inc., West Grove, PA, USA) followed by enhanced chemiluminescence detection (Biological Industries USA Inc., Cromwell, CT, USA).

### Immunohistochemistry

Human osteosarcoma tissues were embedded in paraffin. The slides were blocked with 3% hydrogen peroxide, preincubated in 20% normal goat serum, and probed with anti-SIRT1 followed by biotinylated secondary antibodies and HRP-conjugated avidin. SIRT1 was visualised with 3, 3′-diaminobenzidine.

### Wound healing assay

MDOS-14, MDOS-16, MDOS-19, MDOS-21, MDOS-22, MDOS-26, and MDOS-27 primary osteosarcoma cells were seeded in 24-well plates and cultured to 70–80% confluency. Using a pipette tip, a straight scratch was made to represent an artificial wound. After 24 h, migration of cells across this artificial wound was assessed.

### Cell migration assay

The cell migration assay was performed in a 24-well Transwell plate with 8 μm polycarbonate sterile membranes (Corning Inc., Corning, NY, USA). Cells (2 × 10^4^ cells per insert) were plated in the upper chamber in 200 μL serum-free medium. The inserts were placed in wells containing 600 μL medium supplemented with 10% FBS, and the cells were allowed to migrate for 24 h. At the end of the culture period, the cells on the upper surface were detached with a cotton swab. The filters were fixed in 4% formaldehyde for 10 min, and cells in the lower filter were stained with 0.1% crystal violet for 15 min and counted. The results were calculated by counting three random fields of migrated cells.

### Measurement of *in vivo* activity

Tumours were established via intravenous injection of lentivirus-transfected KHOS/NP cells (1 × 10^5^ cells/animal) into the tail of 3- to 4-week-old female BALB/c (nu/nu) mice (National Rodent Laboratory Animal Resource, Shanghai, China). After the mice were sacrificed, all lungs were dissected and fixed in formalin. Tissue sections (3 μm) were stained with haematoxylin/eosin (H&E). The investigation conforms with the Guide for the Care and Use of Laboratory Animals published by the US National Institutes of Health (NIH Publication No. 85-23, revised 1996). The Animal Research Committee at Zhejiang University approved all animal studies, and animal care was provided in accordance with institutional guidelines.

### Microarray analysis

The microarray analysis was performed as described previously [39], using KHOS/NP cells subjected to GeneChip Human Genome U133 Plus 2.0 Array (Affymetrix, Santa Clara, CA, USA). RNAs were isolated, purified and quantified. Experimental procedures and quality controls for the GeneChip microarray were performed by Gene Tech Co. (Shanghai, China) according to the manufacturer's instructions. Expression levels of all genes were normalised using Partek GS6.5. The one-way analysis of variance (ANOVA) on normalised intensity with a *p*-value ≤ 0.5, followed by ratio change (≥ 2.0), was used to generate the list of genes with significant change. The microarray data of selected probe sets were subjected to cluster analysis using GeneCluster software (UC Berkeley & LBNL; Michael Eisen's lab).

### Statistical analysis

Values for all samples were averaged, and the standard error or standard deviation of the mean was calculated. Differences between means were determined using the one-way analysis of variance (ANOVA), and a *p*-value < 0.05 was considered significant.

## SUPPLEMENTARY FIGURES


