# Supplementary material for: SIRT1 promotes metastasis of human osteosarcoma cells
Source: Oncotarget. 2016 Oct 26;7(48):79654–69. doi: 10.18632/oncotarget.12916 (PMC5346743; doi:10.18632/oncotarget.12916)
Supplement: Supplementary file 1 [file oncotarget-07-79654-s001.pdf]

SIRT1 promotes metastasis of human osteosarcoma cells

SUPPLEMENTARY FIGURES

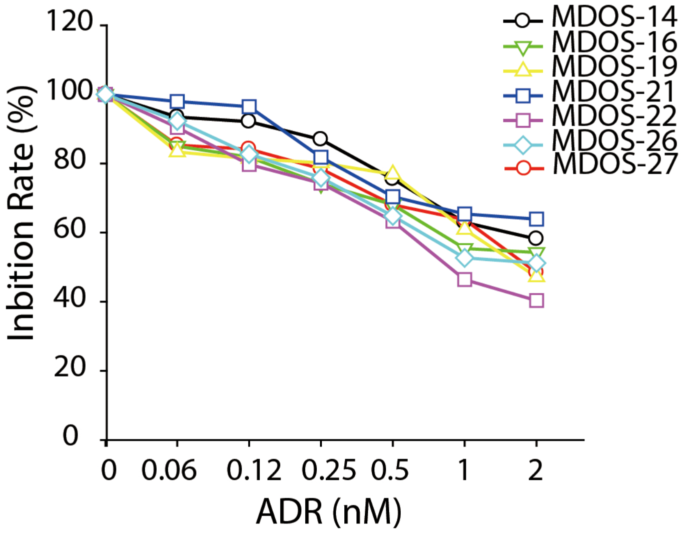

Supplementary Figure S1: The chemotherapy sensitivity of 7 primary osteosarcoma cells to ADR. Primary osteosarcoma cells MDOS-14, MDOS-15, MDOS-16, MDOS-19, MDOS-21, MDOS-22, and MDOS-26 were treated with serial concentrations of ADR for 3 days, and the cytotoxicity was analyzed by SRB assay.

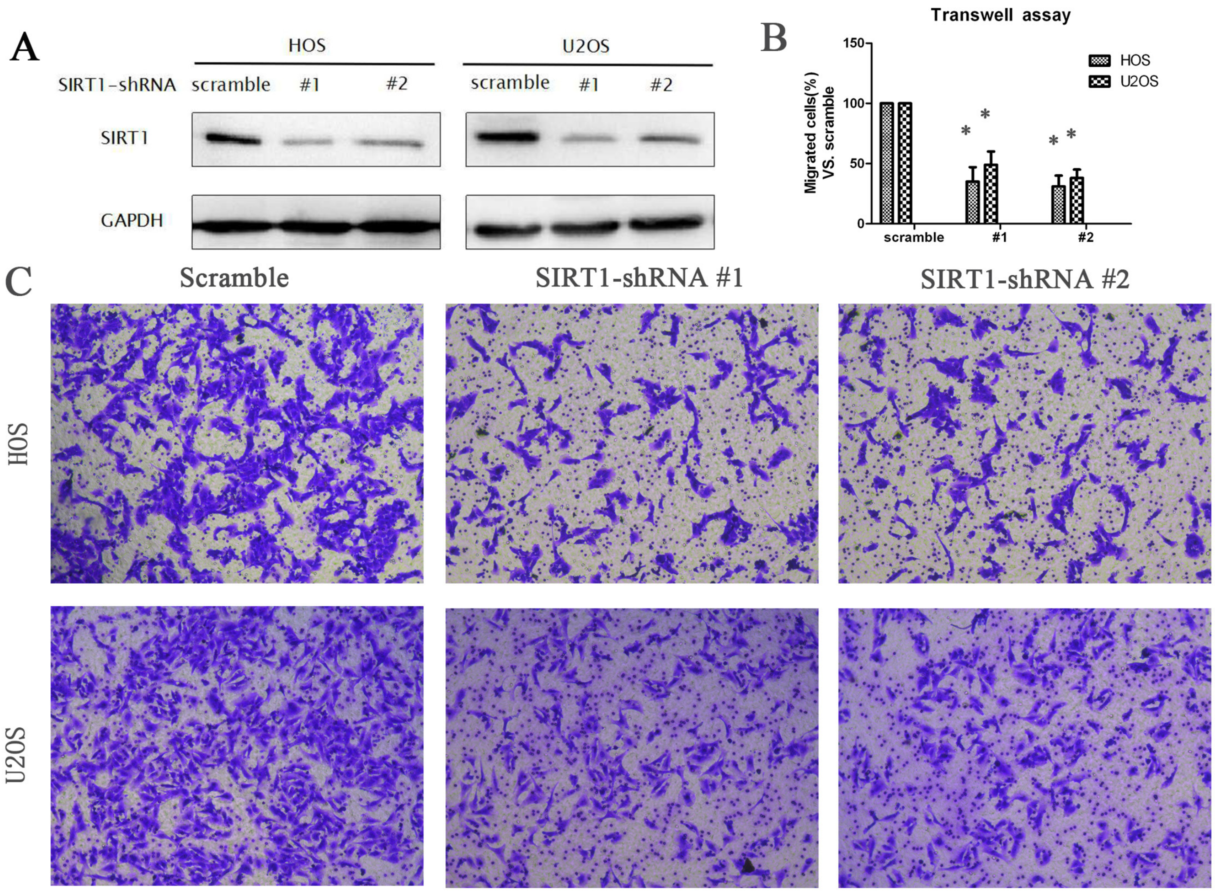

Supplementary Figure S2: Knockdown of SIRT1 inhibits the migration ability of osteosarcoma cell lines HOS and U2OS. A. Western blotting of SIRT1 expression in HOS and U2OS cells after infection with lentivirus- short hairpin RNA (shRNA)-SIRT1 (#1 and #2) or control lentivirus (scramble). **B.** The number of migrated HOS and U2OS cells per field was quantified and shown as a histogram after normalization. Data represent the values triple and are expressed as the means ±SD of three independent experiments. *$P < 0.05$, significant different compared with scramble. 100 × magnification. **C.** Transwell migration assay of HOS and U2OS cells infected with lentivirus-shRNA-SIRT1 (#1 and #2) or control lentivirus (scramble). Representative images of migrated cells are shown.

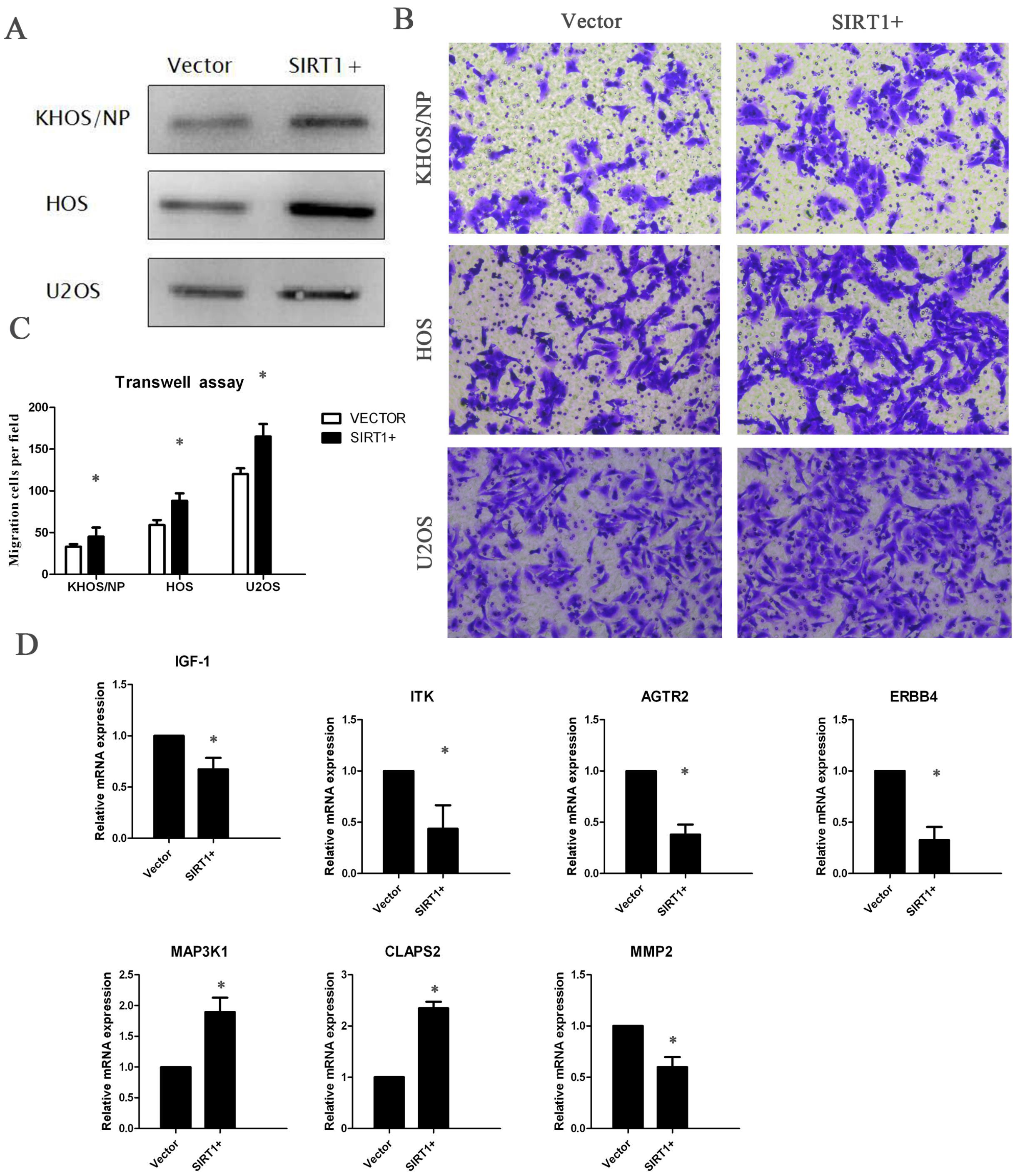

Supplementary Figure S3: Overexpressiong of SIRT1 increases the migration of osteosarcoma cell. A. Western blotting of SIRT1 expression in HOS and U2OS cells after infection with lentivirus or vector. **B.** Transwell migration assay of KHOS/NP, HOS and U2OS cells infected with lentivirus or vector. Representative images were represented. **C.** Migrated cells were counted in three random fields and statistics analyzed. Data are expressed as the means ± SD in triple. *P < 0.05, compared with the vector group. **D.** Real-time PCR examination of gene expressions of IGF-1, ITK, AGTR2, MMP2, ERBB4, MAP3K1 and CLAPS2 in KHOS/NP cells.
